# Contribution of *SLC26A4* to the molecular diagnosis of nonsyndromic prelingual sensorineural hearing loss in a Brazilian cohort

**DOI:** 10.1186/s13104-018-3647-4

**Published:** 2018-08-02

**Authors:** Simone da Costa e Silva Carvalho, Carlos Henrique Paiva Grangeiro, Clarissa Gondim Picanço-Albuquerque, Thaís Oliveira dos Anjos, Greice Andreotti De Molfetta, Wilson Araujo Silva, Victor Evangelista de Faria Ferraz

**Affiliations:** 1 Department of Genetics, Ribeirão Preto Medical School, University of São Paulo, Ribeirão Preto, SP Brazil; 2 Medical Genetics Service of the University Hospital of the Ribeirão Preto Medical School, University of São Paulo, Ribeirão Preto, SP Brazil; 3 Center for Medical Genomics at University Hospital of the Ribeirão Preto Medical School, University of São Paulo, Ribeirão Preto, SP Brazil; 4 Regional Blood Center of Ribeirão Preto (FUNDHERP) of the Ribeirão Preto Medical School, University of São Paulo, Ribeirão Preto, SP Brazil

**Keywords:** Nonsyndromic hearing loss, *SLC26A4*, Mutation screening, DFNB4

## Abstract

**Objective:**

Hereditary hearing loss (HL) is the most common sensorineural disorder in humans. Besides mutations in *GJB2* and *GJB6* genes, pathogenic variants in the *SLC26A4* gene have been reported as a cause of hereditary HL due to its role in the physiology of the inner ear. In this research we wanted to investigate the prevalence of mutations in *SLC26A4* in Brazilian patients with nonsyndromic prelingual sensorineural HL. We applied the high-resolution melting technique to screen 88 DNA samples from unrelated deaf individuals that were previously screened for *GJB2*, *GJB6* and *MT*-*RNR1* mutations.

**Results:**

The frequency of mutations in the *SLC26A4* gene was 28.4%. Two novel mutations were found: p.Ile254Val and p.Asn382Lys. The mutation c.-66C>G (rs17154282) in the promoter region of *SLC26A4*, was the most frequent mutation found and was significantly associated with nonsyndromic prelingual sensorineural HL. After mutations in the *GJB2*, *GJB6* and mitochondrial genes, *SLC26A4* mutations are considered the next most common cause of hereditary HL in Brazilian as well as in other populations, which corroborates with our data. Furthermore, we suggest the inclusion of the *SCL26A4* gene in the investigation of hereditary HL since there was an increase in the frequency of the mutations found, up to 22.7%.

## Introduction

Sensorineural hearing loss (SNHL) is the most common sensorineural impairment in humans, and it is associated with abnormalities of inner ear structures. In most cases, SNHL occurs before speech development (prelingual), and approximately 80% of these cases are affected by mutations in genes related to the hearing process [1, 2]. Many proteins have been associated with hereditary SNHL by affecting the hair-cell structure, extracellular matrix, ion homeostasis, and transcription factors [3, 4]. Mutations in two proteins encoded by the *GJB2* and *GJB6* genes (DFNB1 locus) expressed in cochlea cells, constitute the primary cause of genetic deafness, especially in Caucasian populations [5, 6]. However, studies with the Brazilian population have shown that the screening of the *GJB2* and *GJB6* genes explains the etiology of hearing loss (HL) in only 1–24.7% of the subjects analyzed, being the c.35delG (rs80338939) mutation in *GJB2* (NM_004004.5) gene, the most frequent pathogenic variant [6–9]. Mutations in the *SLC26A4* gene are associated with both syndromic (Pendred Syndrome) and nonsyndromic (DFNB4) cases of SNHL. This gene encodes pendrin, a transmembrane ion transporter which exchanges chloride for iodide and bicarbonate, in the thyroid gland and inner ear. In the cochlea, pendrin is expressed in the epithelial and supporting cells, and it is related to the regulation of pH homeostasis and the ion composition of the endolymph [10, 11]. After mutations in the *GJB2* and *GJB6* genes, mutations in *SLC26A4* are considered the major cause of hereditary hearing loss in the Brazilian population and in many other populations [12–15], contributing to up to 14% of cases of moderate, profound or severe deafness [16]. Thus, this study aimed to investigate and describe the prevalence of mutations in the *SLC26A4* gene in nonsyndromic prelingual sensorineural hearing loss (SNHL) patients of a cohort from the Southeast of Brazil who had been previously tested for *GJB2*, *GJB6,* and *MT*-*RNR1*.

## Main text

### Methods

#### Patient samples and clinical data

This study was approved by the Ethics Research Committee of the University Hospital of the Ribeirão Preto Medical School-USP (n. 8736/2007). Genomic DNAs from 88 unrelated individuals diagnosed with nonsyndromic prelingual SNHL were extracted from whole blood samples, collected on EDTA, using Wizard^®^ Genomic DNA Purification Kit (Promega^®^, Wisconsin, EUA), according to manufacturer’s instructions. To access clinical data, we reviewed the medical archives, and some variables were analyzed, such as type, degree, and progression of hearing loss, familial history, and risk factors. The study enrolled patients of both sexes at an age varying from 2–62 years old, who had been previously tested for *GJB2* mutations by Sanger sequencing of the entire exon; for del(*GJB6*-D13S1830) and del(*GJB6*-D13S1854) deletions in *GJB6* by multiplex PCR; as well as for m.1555A>G (rs267606617) and m.961delT point mutations in the mitochondrial gene *MT*-*RNR1*, by PCR and Restriction Fragment Length Polymorphism (PCR-FRLP) using *HaeIII* (Invitrogen^®^, Wisconsin, EUA) and *MnlI* (Fisher Scientific, New Hampshire, EUA) restriction enzymes.

We used DNA samples from 96 individuals of the same population as a control group. Individuals were all adults, of both sexes and did not present a personal history of hearing loss.

#### Screening of mutations on *SLC26A4* gene

The genomic DNA of the affected individuals was examined for *SLC26A4* mutations based on melting curve analyses compared with wild-type samples using High Resolution Melting (HRM) technique. A total of 26 HRM primers were adapted from Chen et al. [17] to cover all the 21 exons (including the non-coding Exon 1) of *SLC26A4* with a maximum amplicon size of 250pb. The primers were optimized using In-Silico PCR (https://genome.ucsc.edu/cgi-bin/) and checked by the Melting Curve Prediction Software (https://www.dna.utah.edu/umelt/umelt.html) to reach the best melting curve profile (Table 1).

**Table 1:** Primer sequence for amplification of all coding regions of *SLC26A4* gene for HRM mutations analysis

Exon	Amplicon	Primer sequence	Amplicon size (bp)
1	Ex 1	5′-GGGTGGCCCCTGCGTGG-′3	257
		5′-CTCACCTGTCTCTGCTCGC-′3	
2	Ex 2-1	5′-CGTGTCCTCCCTCCTCGCT-′3	113
		5′-GCGCCGCGACACCATGTAG-′3	
	Ex 2-2	5′-GCCGCAGCTCCCCGAGTA-′3	166
		5′-TTCTCTCTACGCAGGCCCGC-′3	
3	Ex 3	5′-GCTTTTTGACAGTTGT-′3	221
		5′-CTATGGTAGCTGGGG-′3	
4	Ex 4-1	5′-TGTAATCACTTTGCATG-′3	99
		5′-GTCAGGATAGGGAAAA-′3	
	Ex 4-2	5′-ATGGTCTCTACTCTGC-′3	93
		5′-GTAAAATATACTTATAATTACC-′3	
5	Ex 5	5′-CTGATTAATTGTTAGAGACTT-′3	218
		5′-CCTGTATAATTCCAACCAG-′3	
6	Ex 6	5′-CGTAGTTGATATTTGGTGGC-′3	248
		5′-GGCCCAGACTCAGAGAATG-′3	
7	Ex 7	5′-GTGCTCGTGTGCGTGTAGC-′3	212
		5′-CTTACCACAATTACTTCTATAGGAA-′3	
8	Ex 8	5′-CATCTTTTGTTTTATTTCAG-′3	125
		5′-CTAAGAGGAACACCACAC-′3	
9	Ex 9	5′-CTAGGTTTTTGCCTCCTGAA-′3	185
		5′-TATAAAACCAGTTCAGCAAAAGG-′3	
10	Ex 10-1	5′-TTGGACCACCACGCAGAG-′3	208
		5′-GACGGCCGTGCGGGAA-′3	
	Ex 10-2	5′-TTCTCTTGTTTTGTGGC-′3	161
		5′-TTGTCCTGCTAAGCTC-′3	
11	Ex 11	5′-CCTTTTCATAGGAGGTGTGTGTC-′3	133
		5′-CGGTATGCAGAGAAGCAGG-′3	
12	Ex 12	5′-CACAGCCTTCTCTGTCT-′3	146
		5′-AATATAGGTGGTAGGTGACT-′3	
13	Ex 13	5′-ATTTTTTTCCCTAGGT-′3	178
		5′-AGGAAGCTCAGAGTGT-′3	
14	Ex 14	5′-TTCCAAAATACGGCTGTTC-′3	154
		5′-AGTCCAGCAAATGTCTCACA-′3	
15	Ex 15	5′-TTGAAATTATTTAATCCCAGACAA-′3	179
		5′-TCTCAAAAGAGGTTAGAAAACAAAT-′3	
16	Ex 16	5′-TTGACATTTATTTCCAA-′3	141
		5′-GGGGGAAAAGAAAGATGTC-′3	
17	Ex 17-1	5′-GACAATTAAGTTGACAGTGTT-′3	182
		5′-GGAACGTTCACTTTGACT-′3	
17	Ex 17-2	5′-GTGGATTGGAACTCTGAGC-′3	150
		5′-GTATAATTCAGAAAACCAGAACC-′3	
18	Ex 18	5′-GAATTATGGGCAGATAAGG-′3	159
		5′-GGCTTACGGGAAAGTCTT-′3	
19	Ex 19-1	5′-TGAGCAATGATGCCAC-′3	247
		5′-AACCTTGACCCTCTTGAG-′3	
	Ex 19-2	5′-GGTTCTTTGACGACAACA-′3	150
		5′-AAAAGATACATCTGTAGAAAG-′3	
20	Ex 20	5′-TGCTATTCTATTTCTACC-′3	142
		5′-TTCAGAAGAAAATGATCAT-′3	
21	Ex 21	5′-ATCAACACTTTGTTTTCC-′3	91
		5′-TATTCCTTGCTCATAGAG-′3	

*Pb* size on base pairs

PCR for HRM analysis was performed on the 7500 Real-Time PCR Systems (Applied Biosystems^®^, California, EUA) using MeltDoctor™ HRM Master Mix (fluorescent DNA intercalating dye) (Applied Biosystems^®^, California, EUA). The reaction mixture in a final volume of 20 μL was made using 10 μL of 1X MeltDoctor™ HRM Master Mix, 2.4 μL of each primer (2.5 nM), 20 ng of genomic DNA and 1.2 μL of dH_2_O. The cycling and melting conditions were as follows: 40 cycles at 95 °C for 15 s, the annealing temperature of each amplicon for 1 min and one cycle at 95 °C for 10 s, 60 °C for 1 min, a melt at 95 °C for 15 s and 60 °C for 15 s. All samples were tested in duplicate.

Samples with mutations identified by HRM analysis were sequenced. Each sample with altered melting curves was purified using the Wizard SV Gel and PCR Clean-up System (Promega^®^, Wisconsin, EUA). Next, the product of purification was sequenced using Sanger sequencing on an automated 3500 XL Genetic Analyzer (Applied Biosystems^®^, California, EUA), using BigDye^®^ Terminator v3.1 Cycle Sequencing Kit (Applied Biosystems^®^, California, EUA) according to the manufacturer´s instructions. Sequencing data were analyzed with the Geneious R7 software v7.1 using the NM_000441.1—GRCh37/hg19 sequence as reference.

In silico analysis of single nucleotide polymorphisms was done using three online tools (Sift Score, PolyPhen-2 and Combined Annotation Dependent Depletion—CADD) to predict the pathogenicity of the non-synonymous variants identified. Then, the software FunSeq and RegulomeDB were used to evaluate the effect of mutations on non-coding regions (exon 1). The Project HOPE Web Server tool was also used to predict the protein structural effects.

For association analyses, we used X^2^ Test and Fisher Exact Test, conducted using R Commander package version 2.4-x with R software environment. p-values lower than 0.05 were considered as a measurement of statistical significance.

### Results

In relation to the degree of hearing impairment, most individuals presented profound (55.7%) or severe (20.4%) hearing loss (HL). About 87.5% presented non-progressive HL, 28.4% reported a positive familial history of deafness and about 13.6% reported parental consanguinity. The frequency of mutations in the *GJB2*, *GJB6,* and mitochondrial genes, was 18.2%. The mutation c.35delG (rs80338939) in the *GJB2* gene was the most frequent, being present in 14 individuals (15.9%) both in homozygous and heterozygous genotypes (Table 2). Three patients presented the del(GJB6-D13S1854) mutation in double heterozygosity with c.35delG in *GJB2*, and two patients presented the del(GJB6-D13S1854) in heterozygosity. No mutation was found in mitochondrial genes (Table 2). However, recessive genotypes and pathogenic mutations that can be associated with the phenotype were found in only 10.2% of the cases.

**Table 2:** *GJB2*, *GJB6* and mitochondrial hotspot mutations previously screened in the 88 unrelated individuals diagnosed with nonsyndromic prelingual SNHL

Sample ID	Previous molecular analysis		
	*GJB2*	*GJB6*	*MT*-*RNR1*
	Coding regions	del(GJB6-D13S1830) del(GJB6-D13S1854)	m.1555A>G m.961delT
2264	c.35delG/*wt*	ND	ND
2282	c.35delG/p.Arg184Trp	ND	ND
2301	c.35delG/*wt*	ND	ND
2671	c.35delG/c.35delG	ND	ND
2768	c.35delG/c.35delG	ND	ND
2778	c.35delG/c.35delG	ND	ND
2853	c.35delG/*wt*	D13S1854/*wt*	ND
2906	c.35delG/*wt*	ND	ND
2966	ND	D13S1830/*wt*	ND
2967	ND	D13S1830/*wt*	ND
3048	c.35delG/c.35delG	ND	ND
3052	c.35delG/w*t*	D13S1854	ND
3067	p.Val27Ile/w*t*	ND	ND
3131	c.35delG/w*t*	D13S1854	ND
3301	c.35delG/c.35delG	ND	ND
3324	p.Arg127Cys/wt	ND	ND

“/wt” means the presence of a wild type allele, i.e. when mutations were found in heterozygosis

*ND* no mutation detected

The inclusion of the *SLC26A4* gene in the molecular screening showed a higher frequency of mutations. Ten non-synonymous mutations were identified in 25 individuals (28.4%). Two mutations were located in the promoter region (exon 1); seven were missense mutations, and two out of seven have not been described yet; as well as this, one synonymous mutation was found. About 22.7% of the mutations were found in heterozygosity or compound heterozygosity and 5.7% in double heterozygosity with the *GJB2* and *GJB6* genes (Table 3). For three of those mutations, it was possible to screen the frequency in the control group of 96 healthy individuals. The most frequent mutation found was the c.-66C>G (rs17154282), located in the non-coding exon 1 of the *SLC26A4* gene (NM_000441.1) and it was found in 14.8% of the cohort. This mutation was significantly associated with nonsyndromic prelingual SNHL patients (OR = 0.33, 95% CI 0.09–1.05, p = 0.03684) when compared to the control group. The novel mutations p.Ile254Val and p.Asn382Lys were found, and this study constitutes the first report on both mutations in prelingual SNHL patients. Both mutations were shown to be pathogenic by the in silico tools PolyPhen-2 and CADD, but we found no significant association of those mutations with SNHL (p = 0.3052) when we compared to the mutation frequency in the control group. Three missense mutations also presented pathogenic scores with the in silico prediction tools (rs111033304; rs36039758 and rs55638457), but they had been previously reported as Benign/Likely benign by the ClinVar database (http://www.ncbi.nlm.nih.gov/clinvar/). No correlations were found between the clinical features and the genotypes.

**Table 3:** Mutations and genotypes found in the *SLC26A4* gene

*SLC26A4* mutations	dbSNP	n^a^	ClinVar status	Pathogenicity scores^b^	Compound heterozygosity	Double heterozygosity			DFNB4 association^c^	
						*GJB2*	*GJB6*	*MT*-*RNR1*	OR (95%CI)	p value
c.-66C>G	rs17154282	13	Benign/Likely benign	2 (FunSeq) 4 (RegulomeDB)	c.-103T>C; p.Asn324Tyr; p.Val609Gly	c.35delG/*wt*; c.35delG/c.35delG	D13S1854	ND	0.33 (0.09–1.05)	p = 0.03684
c.-103T>C	rs60284988	1	Conflicting interpretations of pathogenicity	2 (FunSeq) 5 (RegulomeDB) 17.77 (CADD)	c.-66C>G	ND	ND	ND		
p.Gly5Gly	rs7811324	1	Benign	1.00 (Sift) 22.4 (CADD)	–	ND	ND	ND		
p.Ala189Ser	rs35045430	1	Benign	0.00 (Sift) 1.000 (PolyPhen-2) 13.12 (CADD)	–	ND	ND	ND		
p.Ile254Val	–	1	–	0.62 (Sift) 0.939 (PolyPhen-2) 23.4 (CADD)	–	ND	ND	ND	–	p = 0.3052
p.Ile300Leu	rs111033304	3	Benign/Likely benign	0.00 (Sift) 0.972 (PolyPhen-2) 28.5 (CADD)	–	ND	ND	ND		
p.Asn324Tyr	rs36039758	2	Benign/Likely benign	0.01 (Sift) 0.997 (PolyPhen-2) 26.5 (CADD)	c.-66C>G	ND	ND	ND		
p.Asn382Lys	–	1	–	0.00 (Sift) 1.000 (PolyPhen-2) 31.0 (CADD)	–	ND	ND	ND	–	p = 0.3052
p.Leu597Ser	rs55638457	1	Benign/Likely benign	0.00 (Sift) 0.999 (PolyPhen-2) 29.3 (CADD)	–	c.35delG/c.35delG	ND	ND		
p.Val609Gly	rs17154335	5	Benign/Likely benign	0.54 (Sift) 0.000 (PolyPhen-2) 18.12 (CADD)	c.-66C>G	p.Val27Ile/w*t*	ND	ND		

“/wt” means the presence of a wild type allele, i.e. when the mutations were found in heterozygosis

^a^“n” represents the number of individuals affected by the mutation

^b^The scores will differ from tool to tool. Sift: the mutations are considered as pathogenic when scores ≤ 0.05; PolyPhen-2: the amino acid substitution is considered pathogenic when scores > 0.5; CADD: the mutations are considered pathogenic when PHRED score > 20. FunSeq: scores range from 0 to 6 and as high as the most deleterious. RegulomeDB: scores range from 1a to 6, “1a” harboring the highest number of evidences for each related activity

^c^The analysis of association was performed only for those three mutations. The OR values were calculated in comparison to the frequency of those mutations on the control group, using Fisher’s Exact Test. The p-values were accessed using Pearson’s Chi squared test, both performed by R Commander tool

*ND* no mutation detected

### Discussion

The *SLC26A4* (OMIM 605646) gene encodes pendrin, a protein associated with regulation of the ion composition and pH of endolymph in cochlear cells [11, 18]. Mutations in *SLC26A4* gene may promote pendrin downregulation or loss of function, and constitute the second most frequent cause of autosomal recessive NSHL [4]. In this study, the frequency of mutations in *GJB2*, *GJB6,* and mitochondrial genes was 18.2%, and the inclusion of the *SLC26A4* in the investigation increased the frequency of mutations to up to 40.9%.

Hereditary NSHL is a disease characterized by an autosomal recessive inheritance pattern [3], however, about 18.1% of *SLC26A4* mutations found in our study were monoallelic. These findings corroborate with many studies with NSHL patients [19–21] that report up to 61% of cases presenting heterozygous mutations in the *SLC26A4* gene and suggest that these mutations might contribute to phenotype due to the presence of other mutations not detected (in genes or in regulatory regions not investigated), which together affect pendrin expression in the inner ear. The mutation c.-66C>G (rs17154282) was the most frequent mutation found in our study. It was present in 14.8% (13/88) of the individuals, mainly in a heterozygous genotype. This mutation was also reported by Choi et al. [22] in patients with NSHL and EVA. In the Tunisian population [23], a high frequency of c.-66C>G was also found in patients with autoimmune thyroid diseases, but this was considered as a non-pathogenic polymorphism. The c.-66C>G presents a high allele frequency specially in the African population [24, 25], which may explain the high frequency of that mutation in both the Tunisian and Brazilian populations, once both of whom present a strong African ancestry. In our study, the in silico analyses showed low pathogenicity scores (Table 3) but also suggested that the mutation affects a chromatin regulatory region that is also important for the ligation of enhancers and transcriptional factors (ELF1, RAD21, REST, ZEB1, CTCF). The screening of c.-66C>G in the control group of the same population revealed a significant association of that mutation with hereditary NSHL (OR = 0.33, 95% CI 0.09–1.05, p = 0.03684). Nevertheless, despite the lack of functional evidences, this mutation is reported as Benign/Likely benign on the ClinVar database, probably due to its high allele frequency. Even though there is conflicting data of pathogenicity, our data suggests that c.-66C>G may be related to hereditary NSHL. However, functional assays are necessary to validate the contribution of this mutation to the phenotype.

One case showed c.-66C>G in *trans* with c.-103T>C (rs60284988), which presents a nucleotide change in a conserved region. It was functionally demonstrated [26] that c.-103T>C integrates a critical binding site for transcriptional regulatory elements (as described by the prediction tools cited above), since c.-103T>C affects the binding of FOXI1, and it completely abolishes the FOXI1 activation of *SLC26A4* transcription. Given this case, we suggest that the *SLC26A4* 5′UTR mutations contribute to the phenotype due to the recessive genotype.

Two novel mutations were found in this cohort, but only p.Asn382Lys presented pathogenic scores for all the three in silico prediction tools analyzed (Table 3). None of those mutations were found in the control group, and no significant association with the phenotype was found (p = 0.3052), probably due to the low number of individuals analyzed. The mutations p.Asn324Tyr (rs36039758) and p.Ile300Leu (rs111033304) also presented pathogenic scores but ClinVar also characterizes them as benign. In regard to p.Ile300Leu, this work constitutes the first report in hereditary NSHL [23, 27]. However, segregation analyses were done with a first degree NSHL relative for two of the three probands affected by p.Ile300Leu, and we suggest that this mutation is not related to the phenotype since no segregation of p.Ile300Leu within the NSHL was observed in those families. Segregation analyses were also possible for the p.Val609Gly (rs17154335) mutation, showing no segregation of that mutation within the investigated affected relatives. This corroborates with the results obtained by the prediction tools and many studies that have already characterized p.Val609Gly as a non-pathogenic mutation [19, 20, 28].

### Conclusion

The NSHL is a multigenic complex disease involving many genetic and/or environmental factors [26]. It is known that after mutations in the *GJB2* and *GJB6* genes, *SLC26A4* is considered the next most common cause of hereditary HL in the Brazilian population and in many other populations [12–15]. Our data showed that the inclusion of a single gene in the investigation increased the frequency of mutations from 18.2 to 40.9%, reinforcing the importance of that gene to hereditary hearing loss genetic screening.

## Limitations

Many cases remained with an inconclusive molecular diagnosis, which demonstrates the need for more studies in order to characterize those mutations of unknown significance as well as other non-coding regions and novel genes associated with hereditary hearing loss.

## Abbreviations

CI: confidence interval
DFNB4: neurosensory nonsyndromic recessive deafness 4
EVA: enlarged vestibular aqueduct
HL: hearing loss
NSHL: nonsyndromic hearing loss
OR: odds ratio
PCR: polymerase chain reaction
SNHL: sensorineural hearing loss
SNP: single nucleotide polymorphism

## References

[1] Shearer AE, Hildebrand MS, Smith RJH, Pagon RA, Adam MP, Ardinger HH (1999). Hereditary hearing loss and deafness overview. GeneReviews®.

[2] Lin F, Li D, Wang P, Fan D, De J, Zhu W (2014). Autosomal recessive non-syndromic hearing loss is caused by novel compound heterozygous mutations in TMC1 from a Tibetan Chinese family. Int J Pediatr Otorhinolaryngol.

[3] Van Camp G; Smih R. Hereditary hearing loss homepage, nonsyndromic genes, autosomal recessive. In: Hereditary hearing loss homepage; 2017. http://hereditaryhearingloss.org. Accessed 30 July 2017.

[4] Najmabadi H, Kahrizi K (2014). Genetics of non-syndromic hearing loss in the Middle East. Int J Pediatr Otorhinolaryngol.

[5] Sanchez HA, Verselis VK (2014). Aberrant Cx26 hemichannels and keratitis-ichthyosis-deafness syndrome: insights into syndromic hearing loss. Front Cell Neurosci.

[6] De Freitas Cordeiro-Silva M, Barbosa A, Santiago M, Provetti M, Dettogni RS, Tovar TT (2011). Mutation analysis of GJB2 and GJB6 genes in Southeastern Brazilians with hereditary nonsyndromic deafness. Mol Biol Rep.

[7] Manzoli GN, Abe-Sandes K, Bittles AH, da Silva DSD, Fernandes LDC, Paulon RMC (2013). Non-syndromic hearing impairment in a multi-ethnic population of Northeastern Brazil. Int J Pediatr Otorhinolaryngol.

[8] Manzoli GN, Bademci G, Acosta AX, Félix TM, Cengiz FB, Foster J (2016). Targeted Resequencing of Deafness Genes Reveals a Founder MYO15A Variant in Northeastern Brazil. Ann Hum Genet.

[9] Melo US, Santos S, Cavalcanti HG, Andrade WT, Dantas VG, Rd M (2014). Strategies for genetic study of hearing loss in the Brazilian northeastern region. Int J Mol Epidemiol Genet.

[10] Ammar-Khodja F, Bonnet C, Dahmani M, Ouhab S, Lefevre GM, Ibrahim H (2015). Diversity of the causal genes in hearing impaired Algerian individuals identified by whole exome sequencing. Mol Genet Genom Med.

[11] Dossena S, Bizhanova A, Nofziger C, Bernardinelli E, Ramsauer J, Kopp P (2011). Cellular physiology biochemistry and biochemistr y identification of allelic variants of pendrin (SLC26A4) with loss and gain of function. Cell Physiol Biochem.

[12] Chai Y, Pang X, Chen D, Li L, Chen Y, Sun L (2014). Molecular etiology of non-dominant, hearing impairment in Chinese Hans. Am J Med Genet A.

[13] Svidnicki MCCM, Silva-Costa SM, Ramos PZ, dos Santos NZP, Martins FTA, Castilho AM (2015). Screening of genetic alterations related to non-syndromic hearing loss using MassARRAY iPLEX(R) technology. BMC Med Genet.

[14] De Moraes VCS, Dos Santos NZP, Ramos PZ, Svidnicki MCCM, Castilho AM, Sartorato EL (2013). Molecular analysis of SLC26A4 gene in patients with nonsyndromic hearing loss and EVA: identification of two novel mutations in Brazilian patients. Int J Pediatr Otorhinolaryngol.

[15] de Moraes VCS, Bernardinelli E, Zocal N, Fernandez JA, Nofziger C, Castilho AM (2016). Reduction of cellular expression levels is a common feature of functionally affected pendrin (SLC26A4) protein variants. Mol Med.

[16] Naz S, Imtiaz A, Mujtaba G, Maqsood A, Bashir R, Bukhari I (2017). Genetic causes of moderate to severe hearing loss point to modifiers. Clin Genet.

[17] Chen N, Tranebjærg L, Rendtorff ND, Schrijver I (2011). Mutation analysis of SLC26A4 for pendred syndrome and nonsyndromic hearing loss by high-resolution melting. J Mol Diagn.

[18] Dossena S, Nofziger C, Tamma G, Bernardinelli E, Vanoni S, Nowak C (2011). Molecular and functional characterization of human pendrin and its allelic variants. Cell Physiol Biochem Int J Exp Cell Physiol Biochem Pharmacol.

[19] Pryor SP, Madeo AC, Reynolds JC, Sarlis NJ, Arnos KS, Nance WE (2005). SLC26A4/PDS genotype-phenotype correlation in hearing loss with enlargement of the vestibular aqueduct (EVA): evidence that Pendred syndrome and non-syndromic EVA are distinct clinical and genetic entities. J Med Genet.

[20] Albert S, Blons H, Jonard L, Feldmann D, Chauvin P, Loundon N (2006). SLC26A4 gene is frequently involved in nonsyndromic hearing impairment with enlarged vestibular aqueduct in Caucasian populations. Eur J Hum Genet EJHG.

[21] Wu CC, Lu YC, Chen PJ, Yeh PL, Su YN, Hwu WL (2009). Phenotypic analyses and mutation screening of the SLC26A4 and FOXI1 genes in 101 Taiwanese families with bilateral nonsyndromic enlarged vestibular aqueduct (DFNB4) or pendred syndrome. Audiol Neurotol.

[22] Choi BY, Stewart AK, Madeo AC, Pryor SP, Lenhard S, Kittles R (2009). Hypo-functional SLC26A4 variants associated with nonsyndromic hearing loss and enlargement of the vestibular aqueduct: genotype-phenotype correlation or coincidental polymorphisms?. Hum Mutat.

[23] Kallel R, Niasme-Grare M, Belguith-Maalej S, Mnif M, Abid M, Ayadi H (2013). Screening of SLC26A4 gene in autoimmune thyroid diseases. Int J Immunogenet.

[24] Auton A, Brooks LD, Durbin RM, Garrison EP, Kang HM, 1000 Genomes Project Consortium (2015). A global reference for human genetic variation. Nature.

[25] International HapMap Consortium (2003). The international HapMap project. Nature.

[26] Yang T, Vidarsson H, Rodrigo-Blomqvist S, Rosengren SS, Enerback S, Smith RJH (2007). Transcriptional control of SLC26A4 is involved in Pendred syndrome and nonsyndromic enlargement of vestibular aqueduct (DFNB4). Am J Hum Genet.

[27] Hadj-Kacem H, Kallel R, Belguith-Maalej S, Mnif M, Charfeddine I, Ghorbel A (2010). SLC26A4 variations among Graves’ hyper-functioning thyroid gland. Dis Markers.

[28] Pera A, Dossena S, Rodighiero S, Gandía M, Bottà G, Meyer G (2008). Functional assessment of allelic variants in the SLC26A4 gene involved in Pendred syndrome and nonsyndromic EVA. Proc Natl Acad Sci USA.

